# Comprehensive transcriptomic analysis and meta-analysis identify therapeutic effects of N-acetylcysteine in nonalcoholic fatty liver disease

**DOI:** 10.3389/fphar.2023.1186582

**Published:** 2023-05-15

**Authors:** Keungmo Yang, Hee-Hoon Kim, Young-Ri Shim, Tom Ryu, Chang Wook Kim

**Affiliations:** ^1^ Division of Gastroenterology and Hepatology, Department of Internal Medicine, College of Medicine, The Catholic University of Korea, Seoul, Republic of Korea; ^2^ Life Science Research Institute, Korea Advanced Institute of Science and Technology, Daejeon, Republic of Korea; ^3^ Department of Internal Medicine, Institute for Digestive Research, Digestive Disease Center, Soonchunhyang University College of Medicine, Seoul, Republic of Korea

**Keywords:** nonalcoholic fatty liver disease, N-acetylcysteine, meta-analysis, lipid metabolism, glutathione, inflammation, transcriptomics

## Abstract

**Introduction:** The continuous rise in the prevalence of nonalcoholic fatty liver disease (NAFLD) is emerging as a global health issue. Although the protective effects of N-acetylcysteine (NAC), an antioxidant, against various diseases have been reported, it is still unclear whether NAC has therapeutic potential in NAFLD. Thus, the present meta-analysis aimed to investigate the efficacy of NAC on NAFLD in preclinical studies.

**Methods:** By searching PubMed, Web of Science, and Cochrane Library, 13 studies were included. The methodological quality was assessed based on the SYstematic Review Centre for Laboratory animal Experimentation guideline, and heterogeneity was evaluated with *I* ^2^ and *p* values. Publication bias was assessed by Egger’s test and sensitivity analysis was performed.

**Results:** The results showed that NAC treatment significantly improved systemic and hepatic lipid metabolism (*p* < 0.01), inflammation-related liver injury (*p* < 0.01), glucose intolerance (*p* < 0.05), and hepatic steatosis (*p* < 0.01) by restoring hepatic glutathione (GSH) (*p* < 0.05) and GSH reductase (*p* < 0.05) levels compared to controls in NAFLD-induced animals. Consistently, in bulk, single-cell, and spatial transcriptomics data, the abovementioned target pathways of NAC were strongly associated with NAFLD development in mice and patients.

**Conclusion:** Our study suggests that NAC has therapeutic potential for NAFLD and should be considered for future clinical trials.

## 1 Introduction

Nonalcoholic fatty liver disease (NAFLD) is continuously developed from simple steatosis to inflammation, fibrosis, and hepatocellular carcinoma. Although NAFLD has become the most prevalent chronic liver disease worldwide, still, there is no treatment for licensed except lifestyle intervention which is hard to be achieved (Pouwels et al., 2022). The major challenge in developing drugs for NAFLD is its multiple pathological mechanisms (Buzzetti et al., 2016). In the development of NAFLD, prolonged hypernutrition instigates abnormal expansion in adipose tissue that accompanies low-grade systemic inflammation and lipolysis of adipocytes (Lee et al., 2018). Increased lipolysis in adipocytes elevates circulating levels of free fatty acids (FFAs) that are taken up by hepatocytes expressing fatty acid translocase (FAT/CD36), mediating lipotoxicity that contributes to the generation of hepatic endoplasmic reticulum (ER) stress and oxidative stress (Rada et al., 2020). Otherwise, a series of enzymatic processes by acetyl-CoA carboxylase 1 (ACSL1), monoacylglycerol acyltransferase (MGAT), and diacylglycerol acyltransferase (DGAT) convert FFAs to triglyceride (TG) for storage (Shi and Cheng, 2009; Zeng et al., 2022). Simultaneously, in another hand, western diet (WD) feeding drives gut inflammation and dysbiosis to increase intestinal permeability and hepatic translocation of endotoxins, such as lipopolysaccharides (LPS) (Tripathi et al., 2018). As a consequence of the above multiple hits, both the metabolic arm (lipotoxicity) and inflammatory arm (LPS influx) lead to insulin resistance and liver injury in the pathogenesis of NAFLD.

Recently, the number and scale of clinical trials for NAFLD are rising with its incidence. Indeed, many potential drugs are under phase III clinical trials, including pan-peroxisome proliferator-activated receptor agonist (lanifibranor) (Francque et al., 2021), glucagon-like peptide-1 receptor agonist (semaglutide) (Newsome et al., 2021), thyroid hormone receptor beta agonist (resmetirom) (Harrison et al., 2019), selective farnesoid X receptor agonist (obeticholic acid) (Younossi et al., 2019), a partial inhibitor of hepatic stearoyl-CoA desaturase (aramchol) (Ratziu et al., 2021). Although the above trials have reported some promising effects in lowering liver fat in phase II, there are many adverse effects, such as weight gain, gastrointestinal events, pruritus, and increased low-density lipoprotein (LDL) cholesterol (Dufour et al., 2022). In addition, since these drugs primarily target the metabolic arm of NAFLD pathogenesis and not the inflammatory arm together, it may be hard to achieve histological endpoints as in previous trials. Therefore, new drugs that can cover metabolic and inflammatory arms together are required to combat NAFLD.

N-acetylcysteine (NAC) is a sulfhydryl-containing compound and its medicinal usage has been first reported in 1967 in the prophylaxis of meconium ileus equivalent (Lillibridge et al., 1967). After that, many randomized clinical trials have been conducted and shown the beneficial effects of NAC in drug-induced liver injury, chronic obstructive pulmonary disease, nephropathy, influenza virus infection, and polycystic ovary syndrome (Millea, 2009). A cardinal mechanism of NAC action is restoring the levels of glutathione (GSH), a cellular antioxidant. Interestingly, in addition to this, recent studies have highlighted an anti-inflammatory effect of NAC (Ntamo et al., 2021; Tenorio et al., 2021). In particular, NAC treatment prevents LPS-mediated activation of nuclear factor kappa B, thereby reducing the production of inflammatory cytokines, including tumor necrosis factor (TNF)-α, interleukin (IL)-1β, and IL-6 by macrophages (Tenorio et al., 2021). Moreover, growing evidence from preclinical studies has suggested that NAC also blocks the accumulation of lipids in hepatocytes (Dludla et al., 2020), suggesting that NAC may be a potent therapeutic agent for NAFLD by dual targeting of metabolic and inflammatory arms. However, even though such evidence is accumulating, only small numbers and scales of clinical trials with NAC have been conducted in patients with NAFLD and showed significant reduction in serum alanine aminotransferase (ALT) levels by NAC treatment (Gulbahar et al., 2000; Pamuk and Sonsuz, 2003; Khoshbaten et al., 2010).

Here, to fill the gap between preclinical and clinical studies, we performed a systematic review and meta-analysis for the efficacy of NAC in diverse hepatic or serological markers of NAFLD in a total of 13 preclinical studies (Rosa et al., 2018; Shi et al., 2018; Wang et al., 2018; Chaves Cayuela et al., 2020; Ma et al., 2020; Tsai et al., 2020; Hang et al., 2021; Quesada-Vazquez et al., 2021; Yang et al., 2021; Ding et al., 2022; Lee et al., 2022; Liggett et al., 2022; Yang et al., 2022). In addition, we provide comprehensive transcriptomic data by analyzing bulk, single-cell, and spatial transcriptomics to explore the changes in NAC target pathways in liver tissues of mice and patients with NAFLD and to highlight the therapeutic potential of NAC in NAFLD.

## 2 Materials and methods

### 2.1 Literature searching

Two independent researchers (Keungmo Yang and Hee-Hoon Kim) performed the present meta-analysis with preclinical studies by the preferred reporting items for systematic reviews and meta-analyses (PRISMA) guidelines (Moher et al., 2009). This study was registered in the international prospective register of systematic reviews (PROSPERO) with registration number CRD42023384318. From inception to January 2023, three databases (PubMed, Web of Science, and the Cochrane Library) were utilized to comprehensively search reliable literature about the therapeutic efficacy of NAC for NAFLD. “Acetylcysteine” and “nonalcoholic fatty liver disease” were used as the major keywords, and the variants of words were additionally searched. The detailed search strategy was presented in Supplementary Table S1.

### 2.2 Inclusion and exclusion criteria of literature

The literature searched from the databases was included in the systematic meta-analysis as the following criteria: 1) Animal experiments in all included studies should be approved by the Institutional Animal Care and Use Committee; 2) literature was written in English; 3) animal studies about the efficacy of NAC in NAFLD models; 4) experimental (with NAC treatment) and control (without NAC treatment) groups should be clearly divided in NAFLD-induced animals; 5) studies that presented at least one primary outcome to reflect the effect of NAC in the pathophysiology of NAFLD; 6) NAFLD experimental models should show obvious phenotypic changes in the liver. The studies which had the following criteria were excluded: 1) Duplicated records screened from the different databases; 2) studies that were not related to the efficacy of NAC in NAFLD; 3) systematic reviews, clinical trials, or protocols; 4) conference abstracts; 5) books or theses; 6) lack of data about the primary or secondary outcomes; 7) *in vitro* experiments; 8) literature written in languages other than English.

### 2.3 Data extraction

Elementary data from included studies were extracted after a careful review of two independent researchers. If there were discordances between the extracted data from the two researchers, the final decision was made after the discussion with the third author. The data presented with a graph was analyzed by digitizing software. The detailed factors extracted from the literature are as follows: 1) Publication year and the first author’s name; 2) countries of research institute; 3) animal species and scientific name; 4) sample size (number of animals in each group); 5) animal model to induce NAFLD and specific feeding period; 6) treatment doses, times, and routes of NAC; 7) major outcomes of NAC treatment.

### 2.4 Quality assessment of included studies

The quality of included studies was assessed based on the risk bias tool from the systematic review centre for laboratory animal experimentation (SYRCLE) guideline for animal studies (Hooijmans et al., 2014). The followings are the categories for the quality assessment of the studies: 1) Sequence generation; 2) baseline characteristics; 3) allocation concealment; 4) random housing; 5) blinding of the performance bias; 6) random outcome assessment; 7) blinding of the detection bias; 8) incomplete outcome data; 9) selective outcome data; 10) other sources of bias. The evaluation of each category was divided into “high”, “low”, or “unclear” risk of bias.

### 2.5 Data availability

Bulk RNA sequencing (RNA-seq) or single-cell RNA-seq (scRNA-seq) data from liver tissues of standard diet (SD)- or WD-fed mice are publicly available in the national center for biotechnology information (NCBI) gene expression omnibus under accession number GSE172297 (Zhou et al., 2022) or GSE156059 (Remmerie et al., 2020), respectively. Bulk RNA-seq or spatial transcriptomics data of liver tissues from healthy controls and patients with nonalcoholic fatty liver disease are publicly available in NCBI Gene Expression Omnibus under accession number GSE89632 (Arendt et al., 2015) or GSE192742 (Guilliams et al., 2022), respectively.

### 2.6 Statistical analysis

R software (version 4.2.2; http://cran.r-project.org) and Review Manager (RevMan 5.4) were used for the meta-analysis. The standard mean difference (SMD) and a 95% confidence interval (CI) were analyzed to calculate the treatment efficacy of NAC in NAFLD. The heterogeneity in the included studies was assessed by the *I* ^*2*^ statistic test and Q-square analysis. *I* ^*2*^ > 75% was defined as a high degree of heterogeneity, 75 ≥ *I* ^*2*^ > 25% as a moderate degree of heterogeneity, 25 ≥ *I* ^*2*^ > 0% as a mild degree of heterogeneity, and *I* ^*2*^ = 0% as no heterogeneity. Furthermore, a random-effects model was selected when *I* ^*2*^ > 50% or *p* < 0.1, and a fixed-effects model was selected when *I* ^*2*^ ≤ 50% or *p* ≥ 0.1. A *p*-value <0.05 was considered statistically significant. Publication bias was assessed based on Egger’s linear regression test, and the leave-one-out method was utilized to detect outliers and estimate the sensitivity of the results.

## 3 Results

### 3.1 Identification and selection of study

**FIGURE 1:**
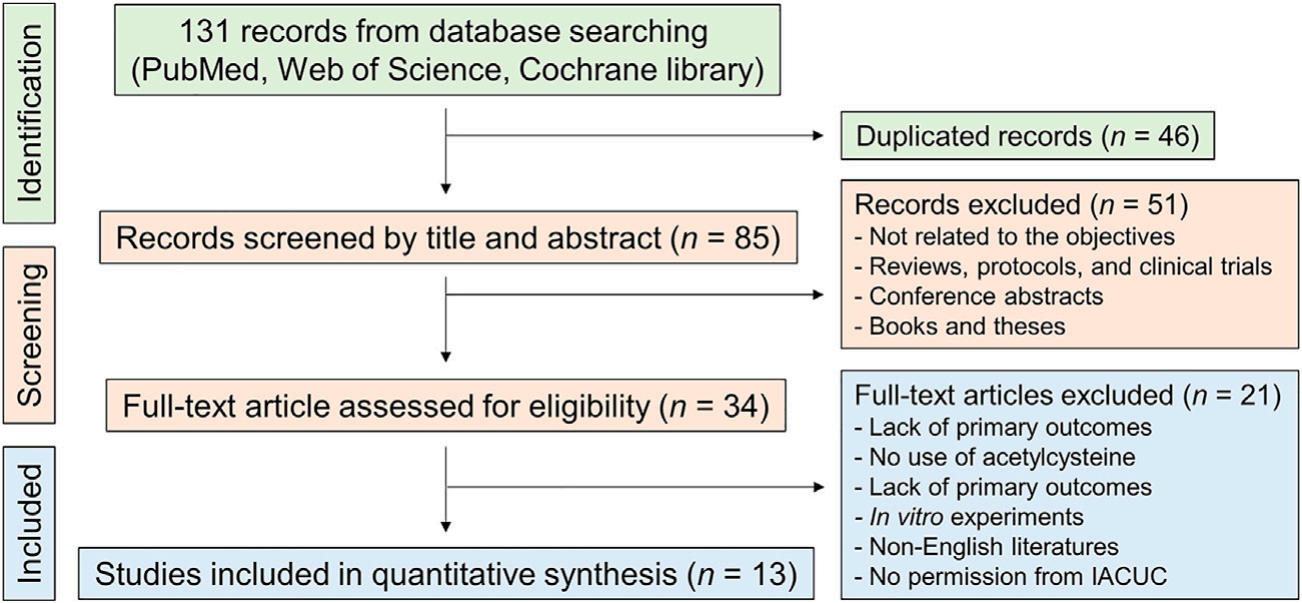
Flowchart for the systematic literature search. IACUC, Institutional animal care and use committee.

According to the literature-searching strategy described in Figure 1, we screened full-text articles for the present meta-analysis. At first, we searched a total of 131 records from three databases (54 from PubMed, 59 from Web of Science, and 18 from Cochrane Library), and 46 duplicated records were removed after the initial screening. After reviewing the titles and abstracts in the records, we excluded the literature that is not related to the efficacy of NAC in NAFLD, systematic reviews, clinical trials, conference abstracts, books, and theses. Then, 34 full-text articles were carefully assessed for eligibility, and a total of 13 studies were finally involved in the meta-analysis (Figure 1) (Rosa et al., 2018; Shi et al., 2018; Wang et al., 2018; Chaves Cayuela et al., 2020; Ma et al., 2020; Tsai et al., 2020; Hang et al., 2021; Quesada-Vazquez et al., 2021; Yang et al., 2021; Ding et al., 2022; Lee et al., 2022; Liggett et al., 2022; Yang et al., 2022).

### 3.2 Study characteristics

The experimental baseline information of the included studies was presented in Table 1. All included articles were published between 2018 and 2022, and the country of the authors was mainly China (46.2%). In terms of animal species, mice (69.2%), rats (23.1%), and hamsters (7.7%) were used for *in vivo* experiments. Various models including a high-fat diet (HFD), methionine-choline deficient (MCD) diet, streptozotocin, or valproic acid were utilized to induce NAFLD in animal experiments. In most studies (84.6%), the route of NAC administration was through oral gavage, and the dose of NAC was varied according to the characteristics of each study. The protective mechanisms of NAC against NAFLD included the reduction of hepatic oxidative stress, improvement in inflammatory liver injury, or decrease in hepatocyte apoptosis (Table 1).

**TABLE 1:** Baseline characteristics of included literature.

Major outcomes	Reduced oxidative stress	Reduced apoptosis of hepatocytes	Reduced inflammation	Reduced oxidative stress and liver injury	Reduced oxidative stress and steatosis	Reduced ER stress and apoptosis	Increased mitochondrial biogenesis	Reduced hepatic steatosis, inflammation, and insulin resistance, but increased β-oxidation		Reduced steatosis, serum cholesterol, glucose, and insulin, but increased HDL cholesterol levels		Reduced free fatty acids and endotoxin levels	Reduced hepatic steatosis, ER stress, apoptosis, and free fatty acid uptake	Reduced hepatic injury	Reduced dyslipidemia, liver injury, and steatosis
Route and times	p.o., daily	p.o., daily	i.p., daily (last 8 weeks)	i.v., single dose (15 min before ischemia)	p.o., every other day	p.o., daily (12 months or first 6 months)	p.o., 7 consecutive days before a high-fat diet	p.o., daily (last 4 weeks)	p.o., daily (last 4 weeks)	p.o., daily (last 4 weeks)		p.o., daily	p.o., daily	p.o., daily	p.o., daily
Dose	25 mg kg^-1^	20, 40, or 80 mg kg^-1^	12.5, 25, or 50 mg kg^-1^	150 mg kg^-1^	200 mg kg^-1^	10 mM in drinking water	150 mg kg^-1^	400 mg kg^-1^ in drinking water		200 mg kg^-1^ in drinking water	400 mg kg^-1^ in drinking water	2 mg mL^-1^ in drinking water	1 mg mL^-1^ in drinking water	In drinking water (doses are not available)	2 mg mL^-1^ in drinking water
Component	N-acetylcysteine	Activated carbon-N-acetylcysteine	N,N’-diacetylcystine	N-acetyl-L-cysteine	N-acetylcysteine	N-acetylcysteine	N-acetylcysteine	Multi-ingredient (400 mg kg^-1^ LCT, 400 mg kg^-1^ N-acetylcysteine, 800 mg kg^-1^ betaine, and 400 mg kg^1^ NAR)		Multi-ingredient (200 mg kg^-1^ LCT, 200 mg kg^-1^ N-acetylcysteine, 400 mg kg^-1^ betaine, and 200 mg kg^1^ NAR)	Multi-ingredient (400 mg kg^-1^ LCT, 400 mg kg^-1^ N-acetylcysteine, 800 mg kg^-1^ betaine, and 400 mg kg^1^ NAR)	N-acetylcysteine	N-acetylcysteine	N-acetylcysteine	N-acetylcysteine
NAFLD model	Streptozotocin (60 mg kg^-1^) (37 days)	High-fat diet (7 weeks)	High-fat diet (20 weeks)	MCD (30 days) + ischemia (30 min)-reperfusion (24 h) injury	Valproic acid (500 mg kg^-1^ day^-1^; 30 days)	High-fat diet (12 months)	High-fat diet (8 weeks)	High-fat diet (12 weeks)	High-fat diet (20 weeks)	High-fat diet (12 weeks)		High-fat diet (12 weeks)	High-fat + cholesterol + cholic acid diet + BPA (50 μg kg^-1^ day^-1^) (8 weeks)	High-fat diet (19–23 weeks)	High-fat diet (14 weeks)
Animal (sex)	Wister rat (male)	Sprague-Dawley rat (male)	Sprague-Dawley rat (male)	C57BL/6J mouse (undefined)	C57BL/6J mouse (male)	C57BL/6J mouse (male)	C57BL/6J mouse (male)	C57BL/6J mouse (male)		Golden Syrian hamster (male)		C57BL/6 mouse (male)	C57BL/6 mouse (male)	C57BL/6 mouse (male)	C57BL/6 mouse (male)
Country	Brazil	China	China	Brazil	China	Taiwan	China	Spain		Spain		China	Taiwan	United States	China
Study	2018 Rosa	2018 Shi	2018 Wang	2020 Cayuela	2020 Ma	2020 Tsai	2021 Hang	2021 Quesada-Vazquez		2021 Yang		2022 Ding	2022 Lee	2022 Liggett	2022 Yang

Abbreviations: BPA, bisphenol A; ER, endoplasmic reticulum; HDL, high-density lipoprotein; LCT, L-carnitine tartrate; MCD, methionine-choline deficient diet; NAR, nicotinamide riboside.

### 3.3 Quality assessment

**FIGURE 2:**
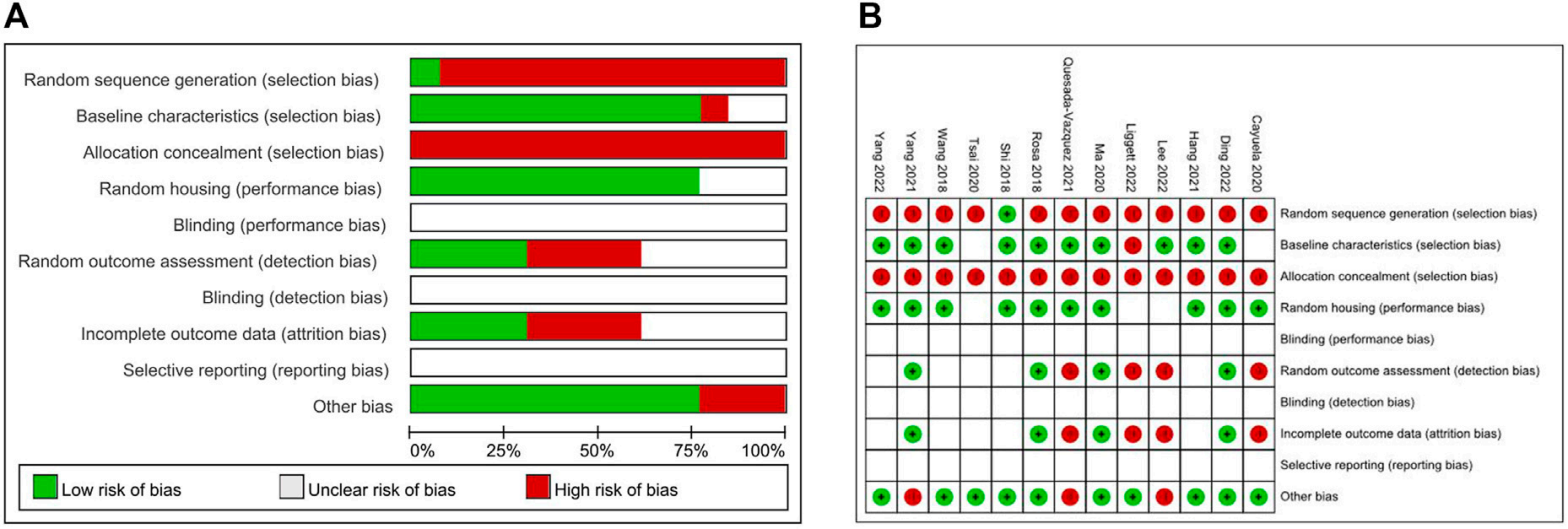
Quality assessment of the included literature. **(A)** The graph represents a risk of bias in included studies. **(B)** Summarizing table for risk of bias of included studies.

The quality assessment of included articles was conducted according to SYRCLE for animal studies (Figure 2A) (Hooijmans et al., 2014). Among the 13 included studies, only one study had a low risk of bias in random sequence generation. In baseline characteristics, 10 studies showed low risk, but one or two studies showed a high or unclear risk of bias, respectively. While all included articles had a high risk of selection bias with allocation concealment, 10 articles showed a low risk of performance bias with random housing. Due to the nature of *in vivo* experiments, it was difficult to estimate the risk of blinding and reporting bias. The pattern of random outcome assessment and incomplete outcome data was the same: 4 studies with high risk, 4 studies with low risk, and 5 studies were unclear of bias. The summary of the risk of bias assessment of each study was depicted in Figure 2B.

### 3.4 NAC treatment mitigates hyperlipidemia in NAFLD-induced animals

**FIGURE 3:**
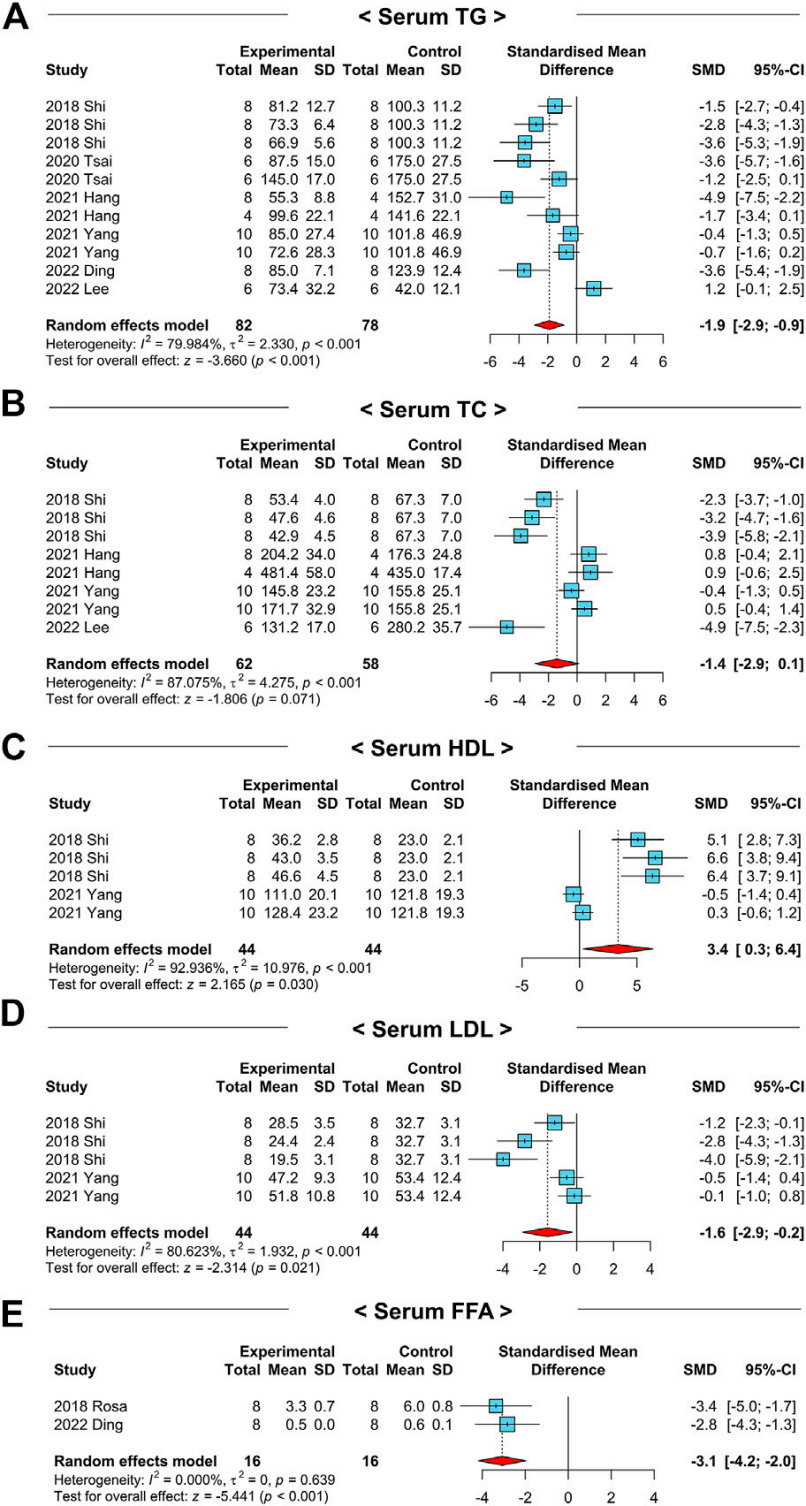
Forest plots for the effects of NAC on markers for systemic lipid profiles in preclinical studies of NAFLD. **(A)** Serum TG levels. **(B)** Serum TC levels. **(C)** Serum HDL levels. **(D)** Serum LDL levels. **(E)** Serum FFA levels. CI, confidence interval; SD, standard deviation; SMD, standardized mean difference.

In the pathophysiology of NAFLD progression, the first step in the “multiple hit” hypothesis is hyperlipidemia caused by persistent nutrient overload (Buzzetti et al., 2016). Therefore, we first evaluated whether NAC treatment could affect systemic or hepatic lipid metabolism in NAFLD-induced animals. In the aspect of serum TG, a total of 6 studies and 160 animals were involved in the analysis (Figure 3A). We performed a random-effects model to calculate the overall effect of NAC because the results had a high homogeneity with a significant *p*-value (*I* ^2^ = 80.0%, *p*-value < 0.01). Notably, serum TG levels were prominently decreased after NAC treatment in NAFLD-induced animals (SMD: −1.9; 95% CI: −2.9 to −1.9 mg dL^-1^; *p*-value < 0.01). Similar to serum TG levels, serum total cholesterol (TC) levels showed a tendency to decrease (SMD: −1.4; 95% CI: −2.9 to −0.1 mg dL^-1^; *p*-value = 0.07) with a high homogeneity between experimental and control groups (*I* ^2^ = 87.1%, *p*-value < 0.01) (Figure 3B). In the publication bias analysis for serum TG levels, there was a substantial asymmetry in funnel plot (Supplementary Figure S1A). Therefore, we performed a sensitivity analysis to examine the effect of each involved study on the overall outcomes. In the leave-one-out analysis, no study affects the beneficial effects of NAC (Supplementary Figure S1B; Supplementary Table S2).

To identify the efficacy of NAC on lipid metabolism in detail, we explored the serum high-density lipoprotein (HDL) and LDL cholesterol levels among the two groups. Interestingly, serum HDL levels were significantly increased (SMD: 3.4; 95% CI: 0.3–6.4 mg dL^-1^; *p*-value = 0.03) while LDL was decreased (SMD: −1.6; 95% CI: −2.9 to −0.2 mg dL^-1^; *p*-value = 0.02) by NAC administration (Figures 3C,D). Furthermore, serum levels of FFAs were remarkably reduced in the NAC-treated group compared to those of the control group (SMD: −3.1; 95% CI: −4.2 to −2.0 mmol L^-1^; *p*-value < 0.01) (Figure 3E).

### 3.5 Administration of NAC reduces hepatic lipid accumulation in NAFLD-induced animals

**FIGURE 4:**
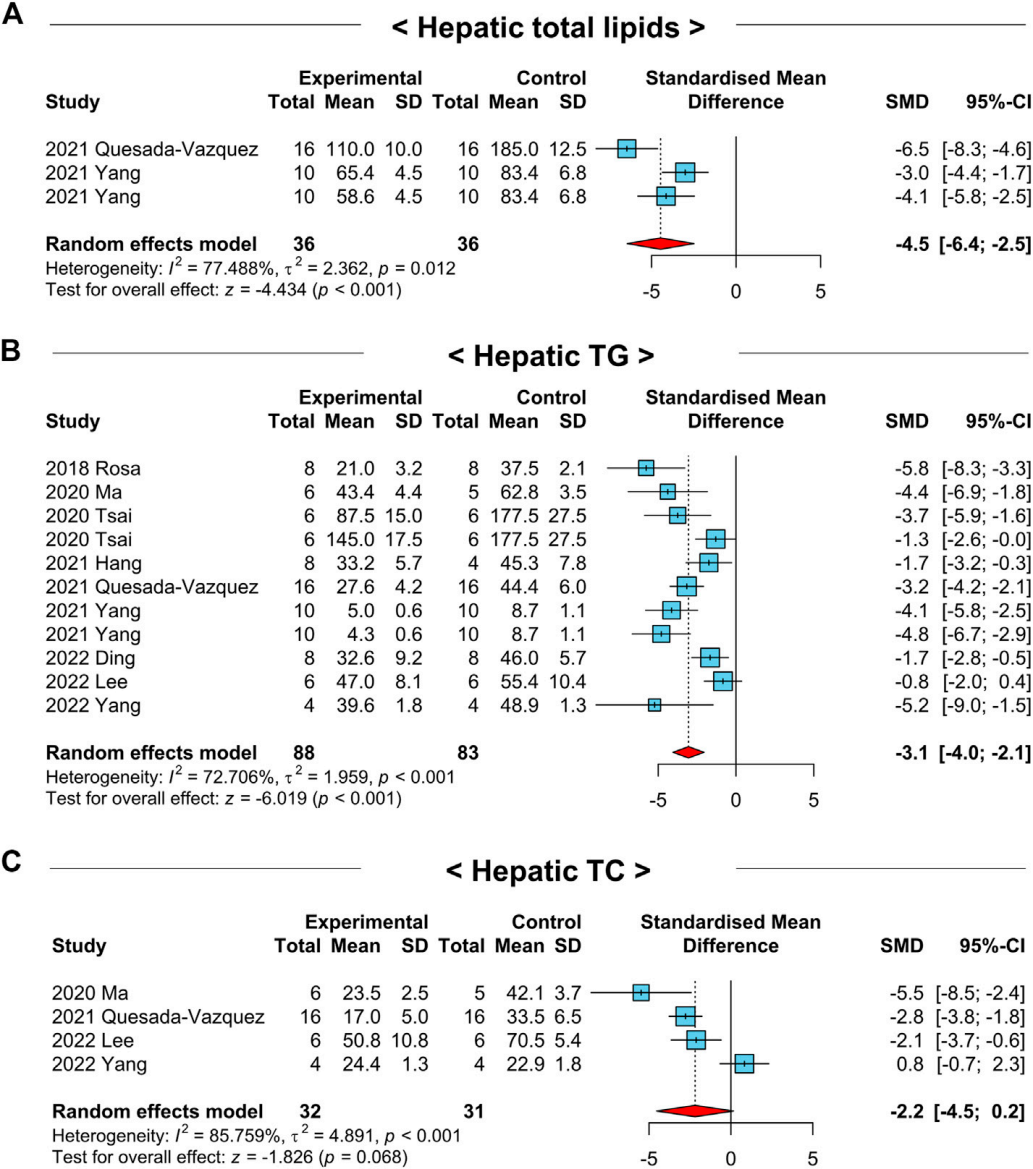
Forest plots for the effects of NAC on hepatic lipid accumulation in preclinical studies of NAFLD. **(A)** Levels of hepatic total lipids. **(B)** Hepatic TG levels. **(C)** Hepatic TC levels. CI, confidence interval; SD, standard deviation; SMD, standardized mean difference.

Next, we examined whether NAC treatment regulates hepatic lipid metabolism in the pathogenesis of NAFLD by analyzing the overall effects of NAC on the markers related to hepatic lipid metabolism in NAFLD-induced animals. We detected significantly decreased hepatic total lipid levels after the treatment of NAC in NAFLD-induced animals (SMD: −4.5; 95% CI: −6.4 to −2.5 mg g^-1^; *p*-value < 0.01) (Figure 4A). To investigate the effects of NAC on hepatic lipid accumulation in detail, we explored the changes in hepatic TG and TC levels by NAC treatment. For hepatic TG levels, a total of 9 studies with 171 animals were included that had a moderate degree of heterogeneity (*I* ^2^ = 72.7%, *p*-value < 0.01) (Figure 4B). In line with decreased hepatic total lipid levels, hepatic TG levels were also significantly diminished by NAC treatment (SMD: −3.1; 95% CI: −4.0 to −2.1 mg g^-1^; *p*-value < 0.01) (Figure 4B). In case of hepatic TC levels, there was a tendency to decrease after NAC administration, although it was not significant (SMD: −2.2; 95% CI: −4.5 to 0.2 mg g^-1^; *p*-value = 0.07) (Figure 4C).

### 3.6 NAFLD-related liver injury is attenuated by NAC treatment in animals

**FIGURE 5:**
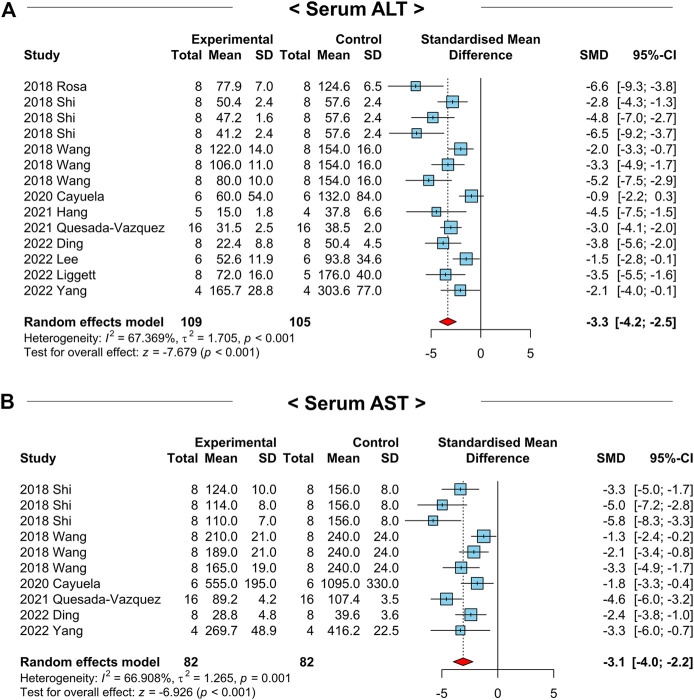
Forest plots for the effects of NAC on markers for liver injury in preclinical studies of NAFLD. **(A)** Serum ALT levels. **(B)** Serum AST levels. CI, confidence interval; SD, standard deviation; SMD, standardized mean difference.

Various hepatic cellular stress (e.g., oxidative stress and ER stress) and inflammatory responses instigate liver injury by exerting hepatocyte death in NAFLD development (Buzzetti et al., 2016). Therefore, we examined whether NAC attenuates NAFLD-related liver injury in preclinical studies by analyzing serum levels of ALT and aspartate aminotransferase (AST). Among the 13 included studies, 10 studies with a total of 214 animals measured the ALT levels. A random-effects model was selected due to a moderate heterogeneity (*I* ^2^ = 67.4%, *p*-value < 0.01) (Figure 5A). As a result, serum ALT levels were remarkably decreased in the NAC-treated groups compared to those of the control groups (SMD: −3.3; 95% CI: −4.2 to −2.5 IU L^-1^; *p*-value < 0.01) (Figure 5A). For the serum AST levels, we investigated 6 studies with 164 animals with a moderate heterogeneity (*I* ^2^ = 66.9%, *p*-value < 0.01) (Figure 5B). Similar to the results from serum ALT levels, NAC treatment significantly diminished serum AST levels in NAFLD-induced animals (SMD: −3.1; 95% CI: −4.0 to −2.2 IU L^-1^; *p*-value < 0.01) (Figure 5B). Collectively, we identified that NAC has a protective effect on the NAFLD-related liver injury in animals.

### 3.7 Suppressive effects of NAC on NAFLD-related systemic inflammation and hepatic apoptosis in animals

**FIGURE 6:**
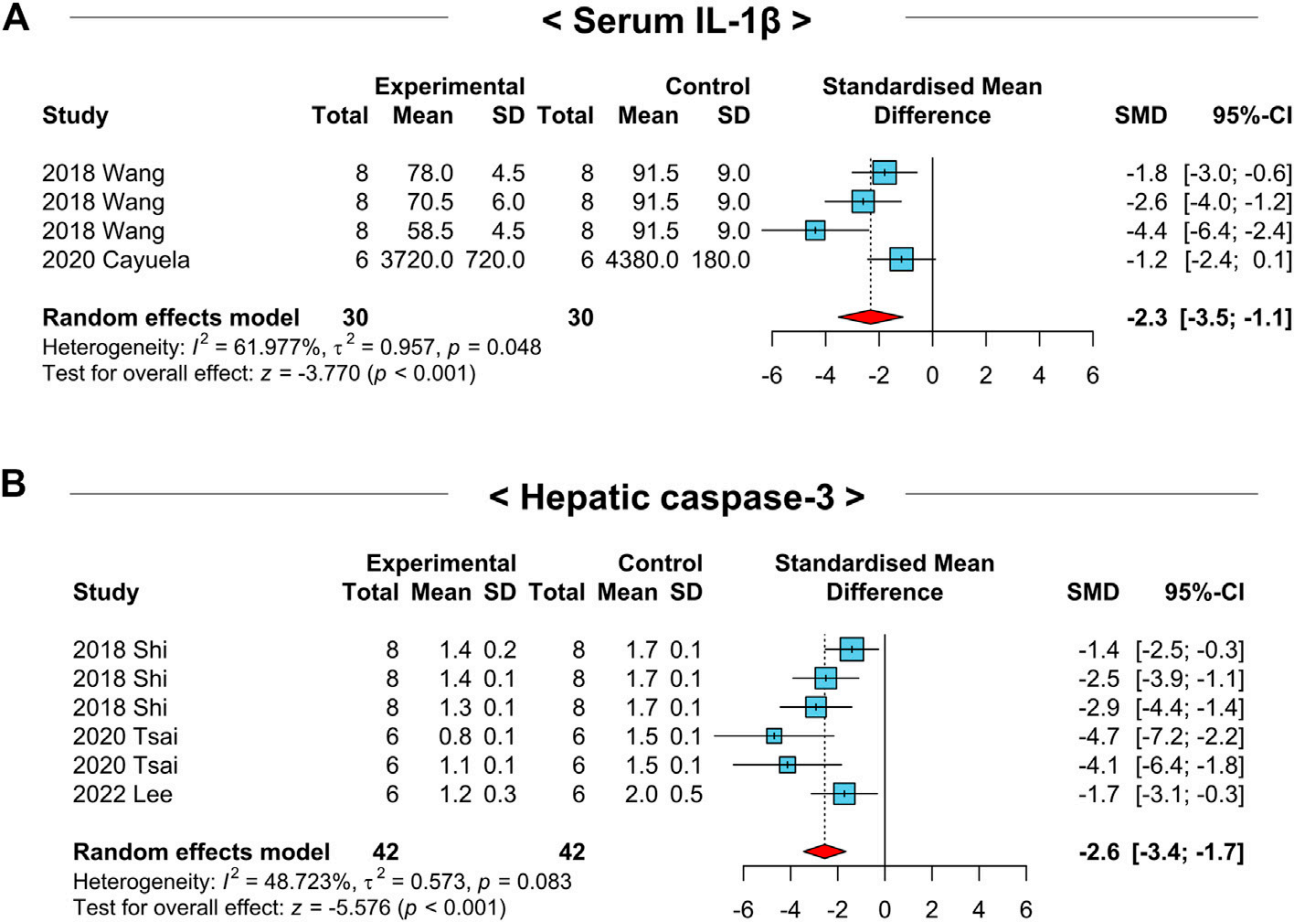
Forest plots for the effects of NAC on markers for systemic inflammation and hepatic apoptosis in preclinical studies of NAFLD. **(A)** Serum IL-1β levels. **(B)** Hepatic caspase-3 levels. CI, confidence interval; SD, standard deviation; SMD, standardized mean difference.

Chronic inflammation is a crucial factor inducing hepatic apoptosis and NAFLD-related liver injury. Thus, we examined the effects of NAC on serum IL-1β and hepatic caspase-3 levels, which are well-defined markers for inflammation and apoptosis, respectively. Interestingly, the serum IL-1β (SMD: −2.3; 95% CI: −3.5 to −1.1 pg mL^-1^; *p*-value < 0.01) and hepatic caspase-3 (SMD: −2.6; 95% CI: −3.4 to −1.7 AU; *p*-value < 0.01) levels were notably reduced in NAFLD-induced animals by NAC treatment (Figures 6A,B). These results may indicate the inhibitory role of NAC on systemic inflammation and hepatic apoptosis.

### 3.8 NAC administration ameliorates glucose intolerance in NAFLD-induced animals

**FIGURE 7:**
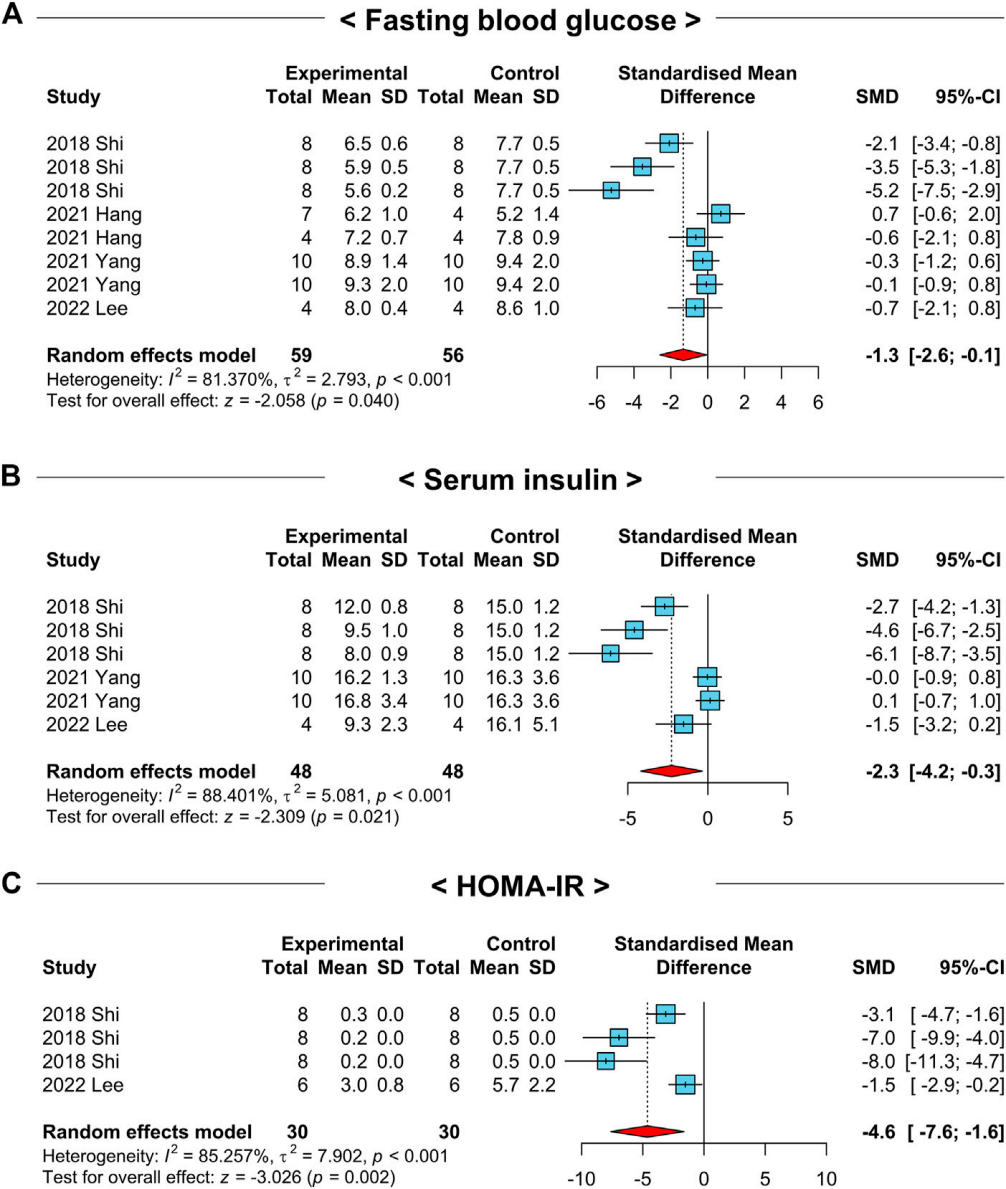
Forest plots for the effects of NAC on insulin resistance in preclinical studies of NAFLD. **(A)** Fasting blood glucose levels. **(B)** Serum insulin levels. **(C)** HOMA-IR. CI, confidence interval; SD, standard deviation; SMD, standardized mean difference.

The development and progression of NAFLD are closely related to obesity, glucose intolerance, and insulin resistance (Pouwels et al., 2022). To explore the efficacy of NAC on the above clinical manifestations, we analyzed the crucial parameters of glucose intolerance in the included studies. First, we investigated the overall effects of NAC on fasting blood glucose levels in 4 studies with 8 comparisons that had a high heterogeneity (*I* ^2^ = 81.4%, *p*-value = 0.04) (Figure 7A). Intriguingly, a random-effects model showed that the treatment of NAC in NAFLD-induced animals significantly reduced fasting blood glucose levels (SMD: −1.3; 95% CI: −2.6 to −0.1 mmol L^-1^; *p*-value = 0.04) (Figure 7A). In addition, NAC administration also declined serum insulin levels in NAFLD-induced animals compared to those of the control groups (SMD: −2.3; 95% CI: −4.2 to −0.3 mU L^-1^; *p*-value = 0.02) (Figure 7B). We additionally investigated the 13 studies that homeostatic model assessment for insulin resistance (HOMA-IR) data was available. Similar to the above findings, HOMA-IR values were notably decreased in NAC-treated groups (SMD: −4.6; 95% CI: −7.6 to −1.6 AU; *p*-value < 0.01) with a strong heterogeneity (*I* ^2^ = 85.3%, *p*-value < 0.01) (Figure 7C). These results indicate that NAC has a beneficial effect on glucose intolerance and insulin resistance in NAFLD-induced animals.

### 3.9 NAC treatment improves histopathological markers of NAFLD-induced animals

**FIGURE 8:**
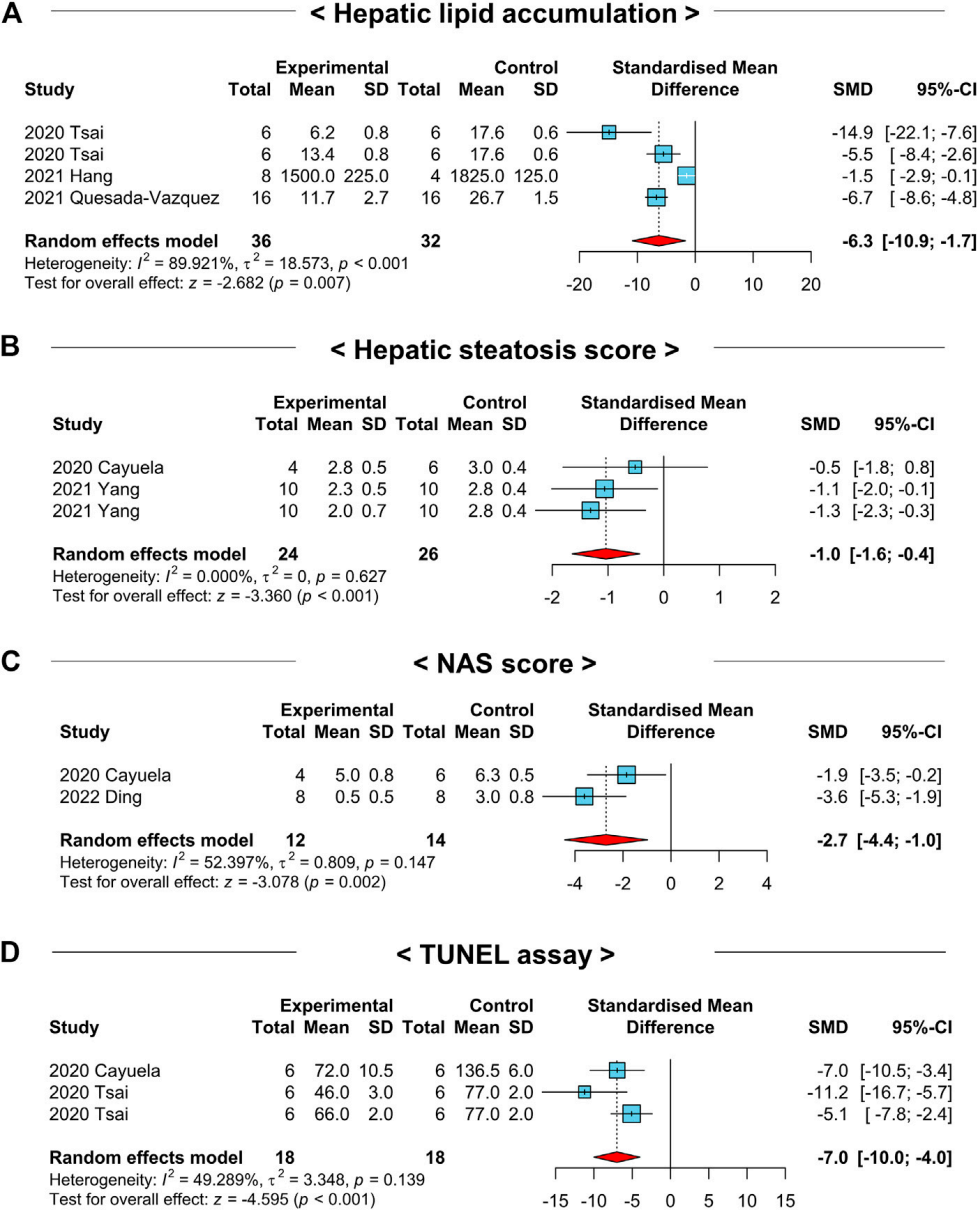
Forest plots for the effects of NAC on liver histological markers in preclinical studies of NAFLD. **(A)** Lipid accumulation in liver tissues. **(B)** Hepatic steatosis score of liver tissues. **(C)** NAS score based on liver tissues. **(D)** TUNEL assay of liver tissues. CI, confidence interval; SD, standard deviation; SMD, standardized mean difference.

Although we detected favorable effects of NAC administration on NAFLD-related systemic or hepatic parameters, it is important to translate the results obtained above into histological findings to cross hurdles in clinical trials. As most NAFLD preclinical studies performed histopathological analysis, we took advantage of this to explore the roles of NAC on histopathological markers in NAFLD-induced animals. Although a small number of studies were analyzed, lipid accumulation in liver tissues measured based on histology was significantly reduced in the NAC-treated groups (SMD: −6.3; 95% CI: −10.9 to −1.7 AU; *p*-value = 0.01) (Figure 8A). Similarly, hepatic steatosis score (SMD: −1.0; 95% CI: −1.6 to −0.4; *p*-value < 0.01) and NAFLD activity score (NAS) (SMD: −2.7; 95% CI: −4.4 to −1.0; *p*-value < 0.01) were prominently mitigated in the NAC-treated animals (Figures 8B,C). Moreover, the analysis of terminal deoxynucleotidyl transferase dUTP nick end labeling (TUNEL) assay in liver tissues indicated significantly fewer apoptotic hepatocytes in the NAC-treated groups compared to those of the control groups (SMD: −7.0; 95% CI: −10.0 to −4.0 AU; *p*-value < 0.01) (Figure 8D). The statistical summary of the overall effects of NAC on lipid metabolism, liver injury, glucose intolerance, and histopathological parameters in NAFLD-induced animals was described in Table 2.

**TABLE 2:** Summary of overall effects of NAC on lipid metabolism, liver injury, glucose intolerance, and liver histopathology in NAFLD-induced mice.

Variable	No. of studies	No. of comparisons	SMD	95% CI	*p*-value of effects	*I* ^2^ (%)	*p*-value of homogeneity
Systemic lipid metabolism							
Serum TG (mg dL^-1^)	6	11	−1.9	−2.9 to −0.9	< 0.01	80.0	< 0.01
Serum TC (mg dL^-1^)	4	8	−1.4	−2.9 to +0.1	0.07	87.1	< 0.01
Serum HDL (mg dL^-1^)	2	5	+3.4	+0.3 to +6.4	0.03	92.9	< 0.01
Serum LDL (mg dL^-1^)	2	5	−1.6	−2.9 to −0.2	0.02	80.6	< 0.01
Serum FFA (mg dL^-1^)	2	2	−3.1	−4.2 to −2.0	< 0.01	0.0	0.64
Hepatic lipid metabolism							
Liver total lipid (mg g^-1^)	2	3	−4.5	−6.4 to −2.5	< 0.01	77.5	0.01
Liver TG (mg g^-1^)	9	11	−3.4	−4.0 to −2.1	< 0.01	72.7	< 0.01
Liver TC (mg g^-1^)	4	4	−2.2	−4.5 to +0.2	0.07	85.8	< 0.01
Liver injury							
ALT (IU L^-1^)	10	14	−3.3	−4.2 to −2.5	< 0.01	67.4	< 0.01
AST (IU L^-1^)	6	10	−3.1	−4.0 to −2.2	< 0.01	66.9	< 0.01
Glucose intolerance							
Fasting blood glucose (mmol L^-1^)	4	8	−1.3	−2.6 to −0.1	0.04	81.4	< 0.01
Serum insulin (mU L^-1^)	3	6	−2.3	−4.2 to −0.3	0.02	88.4	< 0.01
HOMA-IR (AU)	2	4	−4.6	−7.6 to −1.6	< 0.01	85.3	< 0.01
Histopathological parameters							
Lipid accumulation (AU)	3	4	−6.3	−10.9 to −1.7	0.01	89.9	< 0.01
Steatosis score	2	3	−1.0	−1.6 to −0.4	< 0.01	0.0	0.63
NAS score	2	2	−2.7	−4.4 to −1.0	< 0.01	52.4	0.15
TUNEL assay (AU)	2	3	−7.0	−10.0 to −4.0	< 0.01	49.3	0.14

Abbreviations: ALT, alanine aminotransferase; AST, aspartate transaminase; AU, arbitrary unit; CI, confidence interval; HDL, high-density lipoprotein; HOMA-IR, Homeostatic model assessment for insulin resistance; LDL, low-density lipoprotein; NAS, NAFLD, activity score; No., number; SMD, standardized mean difference; TC, total cholesterol; TG, triglyceride; TUNEL, terminal deoxynucleotidyl transferase dUTP, nick end labeling.

### 3.10 Hepatic markers of oxidative stress are improved by NAC treatment in NAFLD-induced animals

**FIGURE 9:**
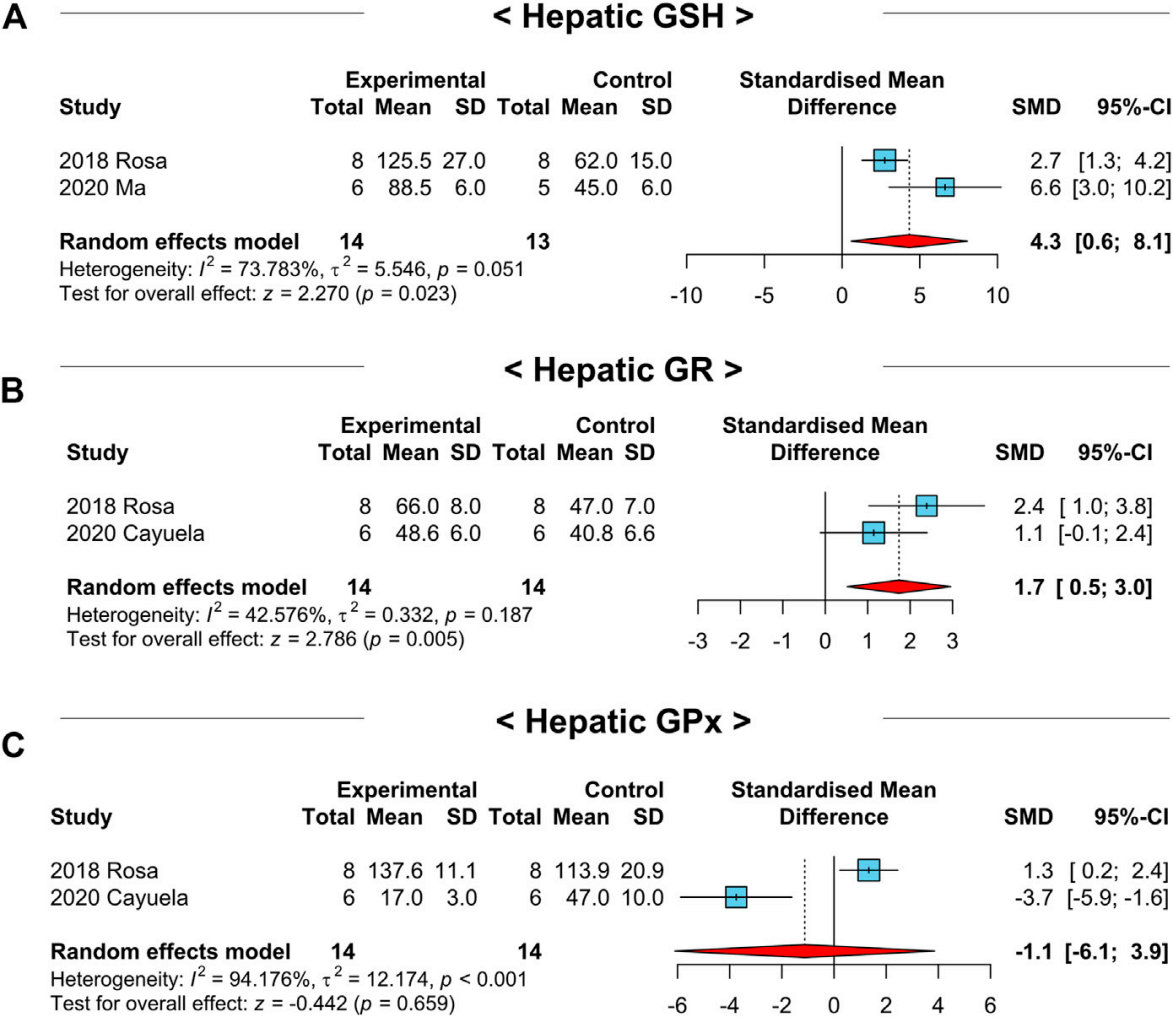
Forest plots for the effects of NAC on markers for hepatic oxidative stress in preclinical studies of NAFLD. **(A)** Hepatic GSH levels. **(B)** Hepatic GR levels. **(C)** Hepatic GPx levels. CI, confidence interval; SD, standard deviation; SMD, standardized mean difference.

It has been reported that NAC restores antioxidant systems in various diseases (Ntamo et al., 2021; Tenorio et al., 2021). Since hepatic oxidative stress exerts inflammatory response and apoptosis of hepatocytes (Tenorio et al., 2021), we tested whether the key factors of the hepatic antioxidant system in the included studies were modulated by NAC treatment. Notably, hepatic GSH (SMD: 4.3; 95% CI: 0.6–8.1 μmol g^-1^; *p*-value = 0.02) and GSH reductase (GR) (SMD: 1.7; 95% CI: 0.5–3.0 μmol mg^-1^; *p*-value = 0.01) levels were significantly increased after NAC treatment (Figures 9A,B). However, hepatic GSH peroxidase (GPx) levels were not differed between the treatment and control groups (SMD: −1.1; 95% CI: −6.1 to 3.9 μmol g^-1^; *p*-value = 0.66) (Figure 9C). In addition, levels of hepatic GSH S-transferase (GST) (SMD: 1.3; 95% CI: −0.8–3.3 μmol mg^-1^; *p*-value = 0.22), catalase (CAT) (SMD: 0.4; 95% CI: −1.4–2.2 μmol mg^-1^; *p*-value = 0.67), and superoxide dismutase (SOD) (SMD: 0.9; 95% CI: −0.6–2.4 nmol mg^-1^; *p*-value = 0.25) were not affected by NAC administration (Supplementary Figures S2A–C). From these findings, we concluded that restoring hepatic GSH levels may be the major pathway to show the antioxidant effect of NAC in NAFLD-induced animals. The summary of the effects of NAC on the hepatic antioxidant system in NAFLD-induced animals was depicted in Table 3.

**TABLE 3:** Summary of overall effects of NAC on hepatic antioxidant system in NAFLD-induced mice.

Variable	No. of studies	No. of comparisons	SMD	95% CI	*p*-value of effects	*I* ^2^ (%)	*p*-value of homogeneity
Hepatic GSH (μmol g^-1^)	2	2	+4.3	+0.6 to +8.1	0.02	73.8	0.05
Hepatic GR (μmol mg^-1^)	2	2	+1.7	+0.5 to +3.0	0.01	42.6	0.19
Hepatic GPx (μmol g^-1^)	2	2	−1.1	−6.1 to +3.9	0.66	94.2	< 0.01
Hepatic GST (μmol mg^-1^)	2	2	+1.3	−0.8 to +3.3	0.22	81.4	0.02
Hepatic CAT (μmol mg^-1^)	2	2	+0.4	−1.4 to +2.2	0.67	80.1	0.03
Hepatic SOD (nmol mg^-1^)	2	2	+0.9	−0.6 to +2.4	0.25	70.9	0.06

Abbreviations: AU, arbitrary unit; CAT, catalase; CI, confidence interval; GPx, glutathione peroxidase; GR, glutathione reductase; GSH, glutathione; GST, glutathione S-transferase; No., number; SMD, standardized mean difference; SOD, superoxide dismutase.

### 3.11 NAC treatment suppresses body weight gain in NAFLD-induced animals

Diet-induced NAFLD experimental models, such as HFD or WD, induce obesity, which has a considerable correlation with the incidence of NAFLD (Lee et al., 2018). To examine whether the above protective effects of NAC against NAFLD are indirectly derived from reduced obesity, we analyzed the body weight changes following NAC treatment in NAFLD-induced animals. Among the analyzed literature, only five studies involved body weight information of used animals that were significantly reduced by NAC treatment (SMD: −2.6; 95% CI: −4.4 to −0.7 g; *p*-value = 0.006) (Supplementary Figure S3).

### 3.12 Comprehensive transcriptomic profiling reveals elevated TG synthesis, inflammatory response, and oxidative stress, but decreased lipid catabolism and insulin resistance pathways in liver tissues of mice and patients with NAFLD

**FIGURE 10:**
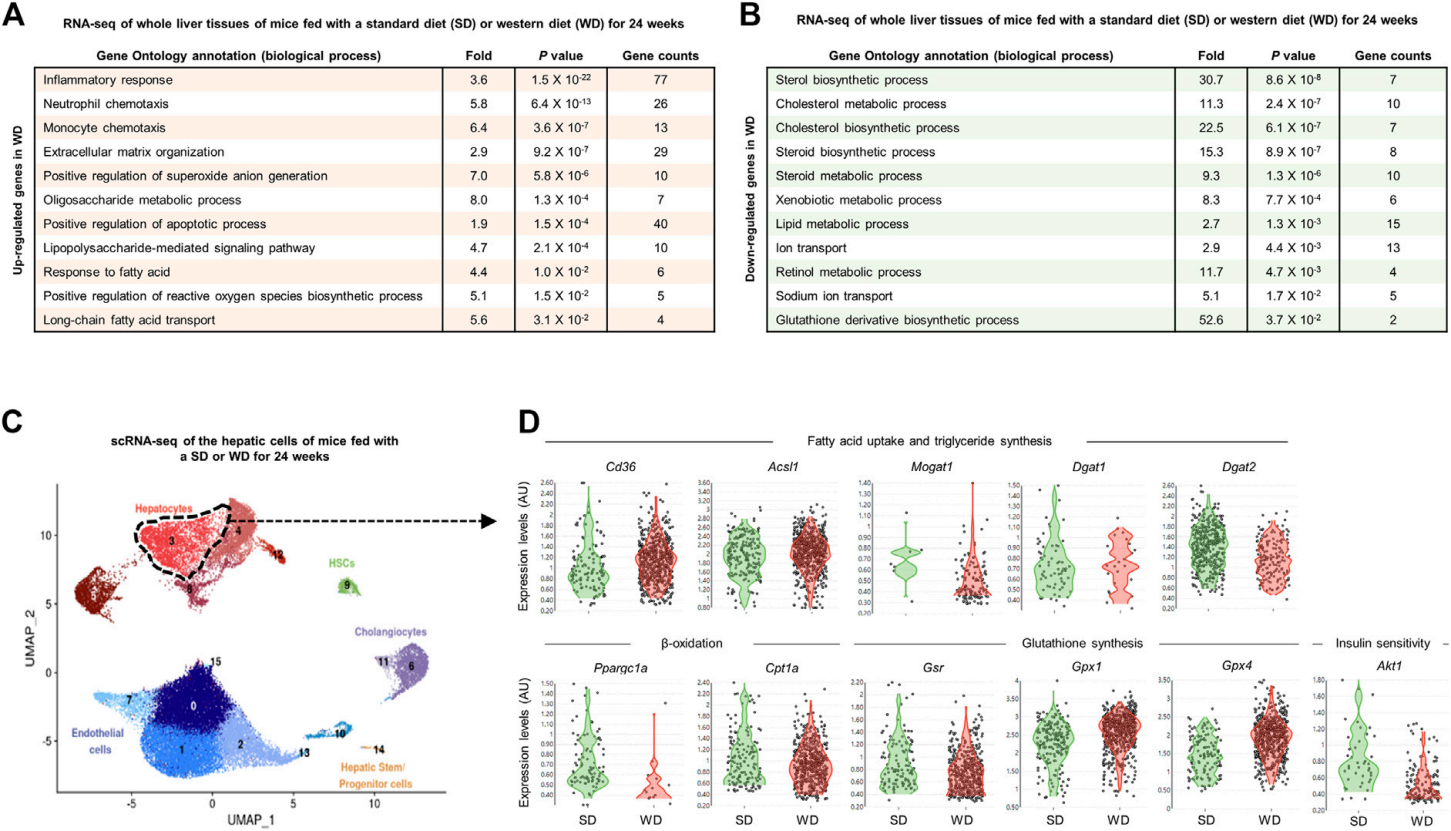
Comprehensive transcriptomic analysis of the liver tissues of mice with WD-induced NAFLD. **(A,B)** Bulk RNA-seq analysis of whole liver tissues of mice fed with a standard diet (SD) or western diet (WD) for 24 weeks (GSE172297). Gene ontology biological processes were analyzed from the list of upregulated **(A)** or downregulated **(B)** genes in WD-fed compared to SD-fed liver tissues. **(C,D)** Single-cell RNA-seq (scRNA-seq) analysis of the hepatic cells of mice fed with SD or WD for 24 weeks (GSE156059). **(C)** Feature plot for cell types presented by uniform manifold approximation and projection (UMAP). **(D)** Violin plots for expression levels of genes related to indicated pathways in hepatocytes (cluster 3). AU, arbitrary unit; UMAP, uniform manifold approximation and projection.

Prompted by the above findings from the meta-analysis of preclinical studies, we next investigated the transcriptional changes in lipid metabolism, inflammatory responses, glucose metabolism, and oxidative stress pathways in NAFLD-induced animals and patients to examine the applicability of NAC to the clinic. Since WD feeding recapitulates the important pathological aspects of patients with NAFLD (Asgharpour et al., 2016), first we analyzed the hepatic transcriptome of WD-fed mice. In the bulk RNA-seq analysis of whole liver tissues of mice fed with a SD or WD for 24 weeks (Zhou et al., 2022), we found notably upregulated biological processes related to inflammatory response, extracellular matrix organization, positive regulation of reactive oxygen species biosynthesis and apoptotic process, and fatty acid transport (Figure 10A). Conversely, expression levels of genes associated with cholesterol, lipid, or retinol metabolism and GSH derivative biosynthetic process were significantly diminished by WD feeding (Figure 10B). In line with this, scRNA-seq analysis of hepatic cells of mice fed with a SD or WD for 24 weeks (Remmerie et al., 2020) identified that expression levels of genes related to FFA uptake (*Cd36*), TG synthesis (*Acsl1, Dgat1,* and *Dgat2*), and GPx (*Gpx1* and *Gpx4*) were increased, while β-oxidation (*Ppargc1a* and *Cpt1a*), GR (*Gsr*), and insulin signaling (*Akt1*) were decreased and *Mogat1* was not changed in hepatocytes of NAFLD-induced mice (Figures 10C,D).

**FIGURE 11:**
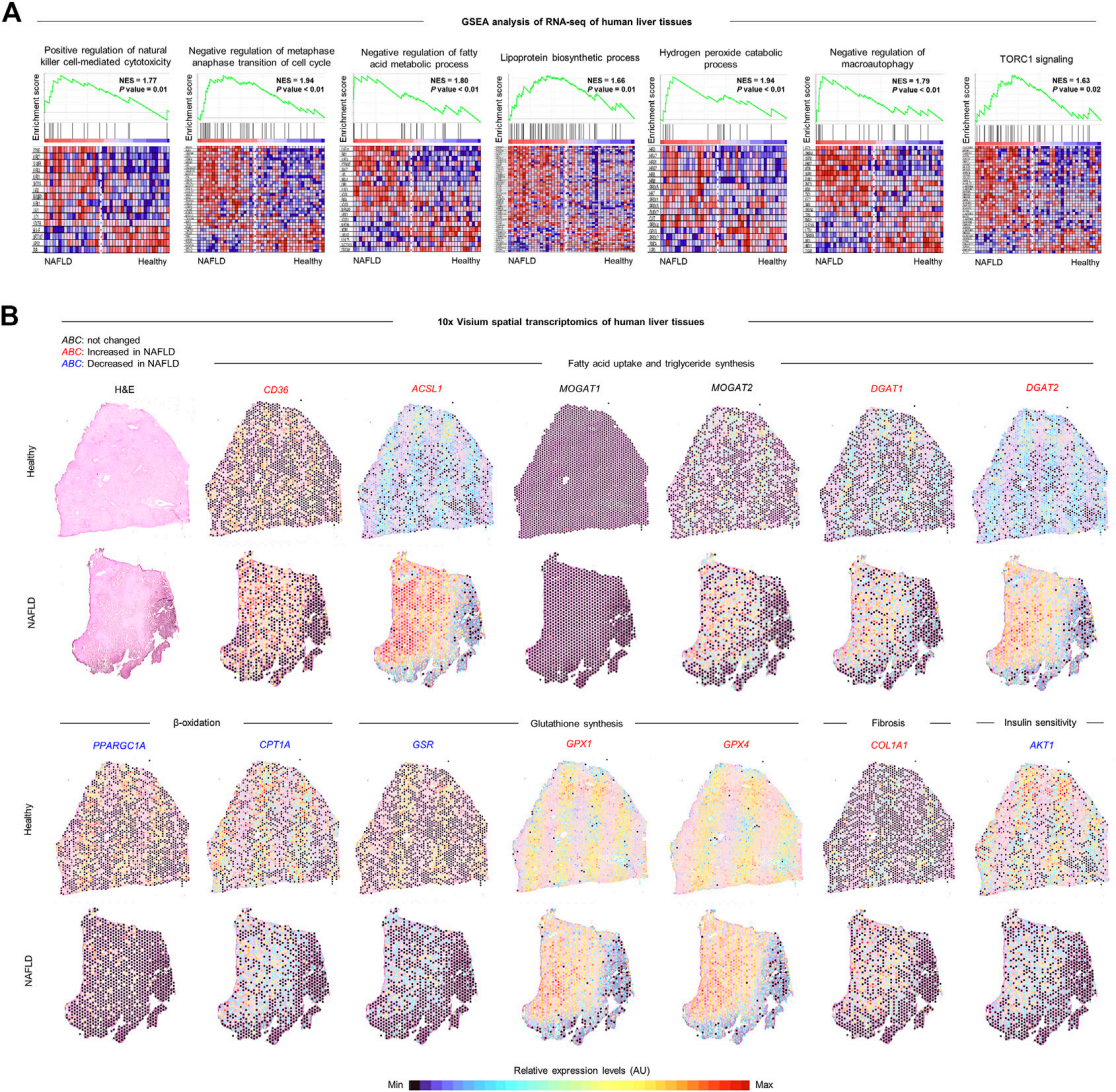
Comprehensive transcriptomic analysis of the liver tissues of patients with NAFLD. **(A)** Geneset enrichment analysis (GSEA) of bulk RNA-seq of whole liver tissues of healthy controls (*n* = 24) and patients with NAFLD (*n* = 20) (GSE89632). Normalized enrichment score (NES). **(B)** 10x Visium spatial transcriptomics analysis of liver tissues of healthy controls and patients with NAFLD (GSE192742). Each plot represents expression levels of genes related to indicated pathways depending on the region. Colors of gene symbols show that expression levels are similar (black), increased (red), or decreased (blue) in NAFLD compared to those of healthy control. AU, arbitrary unit.

Next, we explored the changes in hepatic transcriptional profiles of patients with NAFLD. Consistent with the findings from NAFLD-induced mice, expression levels of genes related to positive regulation of natural killer cell-mediated cytotoxicity, negative regulation of metaphase anaphase transition of cell cycle, negative regulation of fatty acid metabolic process, lipoprotein biosynthetic process, hydrogen peroxide catabolic process, negative regulation of macroautophagy, and TORC1 signaling were enriched in liver tissues of patients with NAFLD compared to those of healthy controls in the gene set enrichment analysis (GSEA) of RNA-seq analysis (Figure 11A) (Arendt et al., 2015). In addition, 10x Visium spatial transcriptomic analysis (Guilliams et al., 2022) revealed that expression levels of genes associated with FFA uptake (*CD36*) and TG synthesis (*ASCL1, MOGAT2, DGAT1,* and *DGAT2*), GPx (*GPX1* and *GPX4*), and fibrosis (*COL1A1*) were increased, while β-oxidation (*PPARGC1A* and *CPT1A*), GR (*GSR*), and insulin signaling (*AKT1*) were reduced and *MOGAT1* was rarely expressed in the liver tissues of patients with NAFLD compared to those of healthy controls, particularly around the lipid-laden hepatocytes (Figure 11B). Together, these data indicate that NAC-targeted pathways are upregulated in the liver tissues of patients with NAFLD, reinforcing the possible application of NAC to the clinic.

## 4 Discussion

**FIGURE 12:**
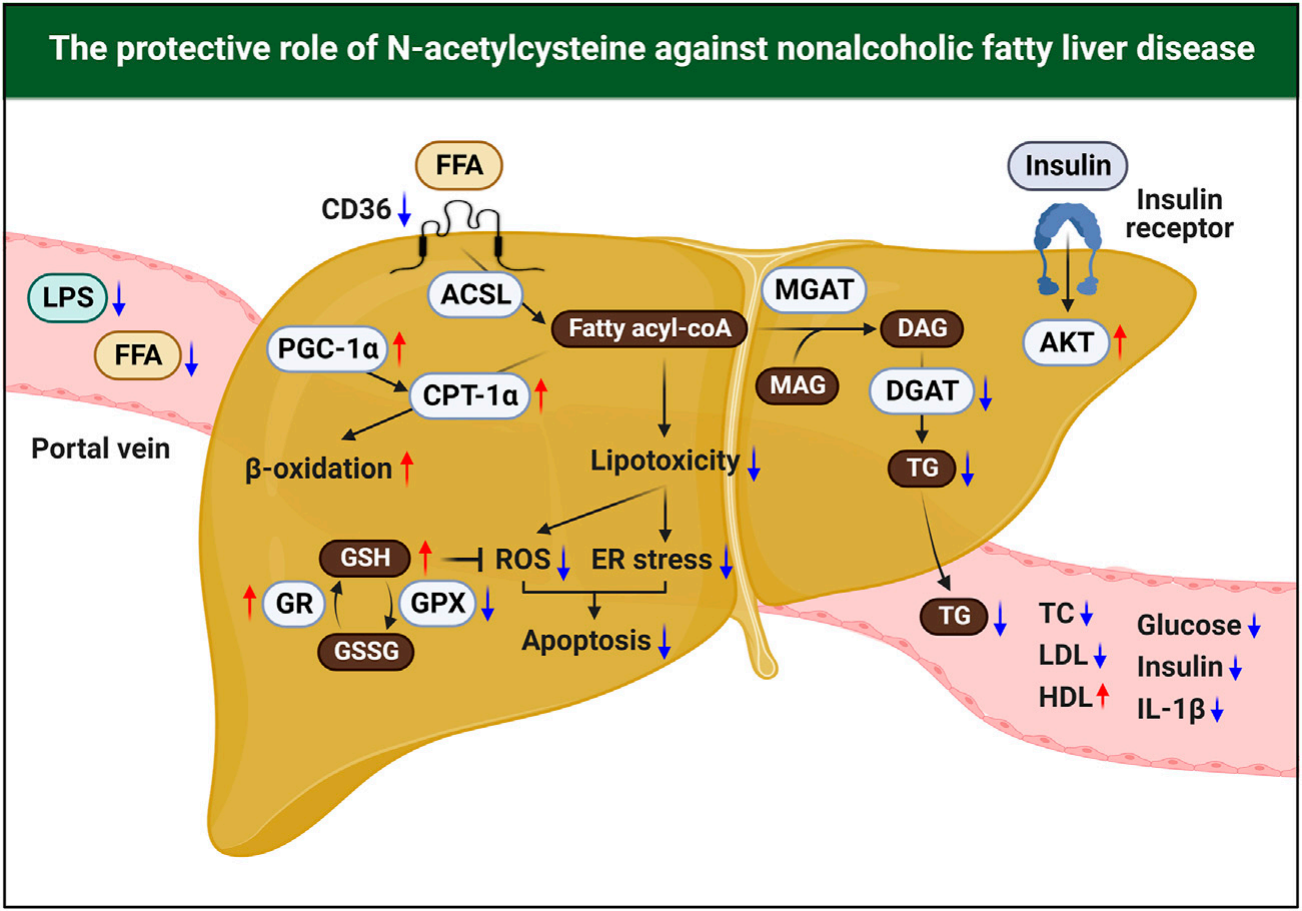
Schematic summary for the therapeutic effects of NAC in NAFLD.

Currently, although the incidence of NAFLD has been increasing worldwide, there is no licensed drug for NAFLD (Pouwels et al., 2022). In the present meta-analysis, we found that diverse doses and duration of NAC treatment significantly attenuated the levels of NAFLD-related pathological markers and histological pathology scores in preclinical studies. In addition, we systematically reviewed the protective mechanisms of NAC against NAFLD. The pathogenesis of NAFLD involves metabolic and inflammatory alterations both inside and outside of the liver. In particular, persistent energy surplus elevates intrahepatic influx of FFA or LPS from adipose tissue or intestine, respectively. In hepatocytes, accumulated FFA exerts oxidative stress and ER stress, resulting in hepatic injury together with LPS-induced inflammation in NAFLD (Buzzetti et al., 2016). Unlike previous drugs currently undergoing clinical trials that target either metabolic or inflammatory processes, our findings suggested that NAC could cover both the metabolic and inflammatory arms of NAFLD pathogenesis, such as dyslipidemia, hepatocyte death, and insulin resistance, at least in rodents (Figure 12). Moreover, we validated and highlighted our findings by comprehensively analyzing bulk, single-cell, and spatial transcriptomics datasets from the liver tissues of mice and patients with NAFLD. Interestingly, we observed similar findings in mice and patients with NAFLD, including increased TG synthesis but decreased β-oxidation, oxidative stress with GSH shortage, inflammatory response, and insulin resistance, all of which are involved in the NAC target pathways. All these results may indicate that NAC is a potent and effective drug for treating NAFLD.

Despite the above promising results from preclinical studies, only a few small scales of NAFLD clinical trials have been conducted with NAC. In 2000, the first uncontrolled study by Glubahar et al. showed a significant reduction in serum ALT, AST, and gamma-glutamyl transferase (GGT) levels by 3 months of NAC (1 g per day) treatment compared to those of baseline in 11 NASH patients (Gulbahar et al., 2000). After that, Pamuk et al. performed experiments that consisted of a total of 30 patients, 15 patients each for the control or NAC group. In this study, the authors daily and orally administered 600 mg of NAC for 4 weeks, and found a significant reduction in serum ALT levels in both groups; however, serum AST and GGT levels were only decreased in the NAC group (Pamuk and Sonsuz, 2003). In addition, Khoshbaten et al. examined the efficacy of 3 months of NAC (600 mg per 12 h) treatment in 15 NAFLD patients and found significantly decreased serum ALT levels by NAC treatment (Khoshbaten et al., 2010). Moreover, 12 months of combinatorial oral administration of NAC (1.2 g per day) and metformin (850–1,500 mg per day) notably improved histological parameters, including hepatic steatosis, ballooning, and NAS score with decreased serum ALT levels in NASH patients (de Oliveira et al., 2008; Oliveira et al., 2019). All these results suggest a protective role of NAC in NAFLD and the use of NAC alone or as a combination treatment in the clinic.

Previous studies have reported that the administration of NAC suppresses cellular oxidative stress by restoring GSH levels (Ntamo et al., 2021). Consistent with this, we found notably increased hepatic GSH levels in NAC-treated animals with upregulated GR activity. However, although hepatic GPx activity was a tendency to decrease by NAC treatment in animals, expression levels of hepatic *GPX1* and *GPX4* were higher in mice and patients with NAFLD compared to those of controls in transcriptomic analyses. This discrepancy may be derived from the small numbers (only two studies) of included studies in analyzing the effects of NAC on hepatic GPx activity. Another study has suggested that systemic NAC treatment normalized enzymatic activities of other cellular oxidants, such as SOD and CAT, in serum or parotid glands of HFD-fed rats (Zukowski et al., 2018). However, we could not see significant changes in the hepatic SOD or CAT enzymatic activities since our meta-analysis only included two studies with 14 animals for these parameters. Therefore, further study is required to demonstrate the effects of NAC on hepatic SOD and CAT activities.

Since it is hard to get the liver tissues from NAFLD patients, most of the clinical trials used serological markers (e.g., serum ALT, AST, TG, TC, HDL, LDL, FFA, glucose, and insulin levels) for the primary outcomes. In line with this, we carefully selected the preclinical studies that investigated similar primary outcomes for meta-analysis so that the results can be well reflected in the clinic. In addition, in our meta-analysis, we found a remarkable improvement in histological scores by NAC treatment in NAFLD-induced animals although the number of included studies is small. Among the growing number of clinical trials for NAFLD, only a few drugs have entered phase III trials, and most have historically failed because they could not meet histological outcomes in patients with NAFLD (Dufour et al., 2022). In this aspect, our results could be a headstone for a compelling advance in NAFLD treatment. Moreover, our study has a particular clinical significance in that we showed a correlation of NAC target mechanisms with pathological changes in animal models and patients with NAFLD at the transcriptional level.

Our study also has several limitations. First, although we provided a comprehensive transcriptomic analysis of NAFLD patients, our meta-analysis was conducted sorely based on the preclinical studies with NAC. Considering the characteristic of animal studies, our results might have a higher heterogeneity compared to randomized controlled trials in human NAFLD patients. In particular, most of the included articles in the present meta-analysis used only HFD to induce NAFLD. Therefore, there may be some differences in the NAFLD pathogenesis compared to patients. Second, some of the analyzed studies that used the HFD-induced NAFLD experimental model in this meta-analysis showed reduced body weight following NAC treatment. These results may suggest the possibility that the beneficial effects of NAC in hepatic lipid metabolism of the NAFLD-induced animals were indirectly derived from the extrahepatic (e.g., adipose tissue) role of NAC. Third, we found that all of the 13 included studies were at risk of bias based on the SYRCLE’s guideline (Hooijmans et al., 2014), and had methodological limitations in quality assessment. Fourth, in the present study, we could not define the effective doses, duration, and route of NAC for treating NAFLD. Despite our findings indicating the protective roles of NAC in NAFLD in animals, it is very hard to define the appropriate setting of drugs for patients based on these findings. In addition, although previous studies have reported minimal adverse effects at 1 g per day of NAC in NAFLD patients (Gulbahar et al., 2000; Pamuk and Sonsuz, 2003; Khoshbaten et al., 2010), a combinatorial treatment of NAC with granulocyte colony-stimulating factor (G-CSF) showed higher mortality than G-CSF alone in patients with severe alcohol-associated hepatitis (Singh et al., 2018), suggesting unknown harmful effects of NAC depending on the microenvironment. Lastly, there was a considerable publication bias in involved studies for serum TG levels. Even though the sensitivity analysis showed that the TG-lowering effect of NAC was not affected by a single study, the results should be interpreted with caution.

The above findings and limitations of the present study provide future directions. First, to demonstrate our findings and apply them to the clinic, more well-designed animal experiments are required. In particular, it might be better to use WD as a NAFLD model instead of HFD because WD-induced NAFLD more resembles the pathological features of NAFLD patients than those of HFD (Asgharpour et al., 2016). In addition, future preclinical studies with NAC have to follow SYRCLE’s and animal research: reporting of *in vivo* experiments (ARRIVE) guidelines to obtain more reliable and reproducible results (Hooijmans et al., 2014; Percie du Sert et al., 2020). Second, large-scale studies on the efficacy and safety of various doses of NAC should be conducted in NAFLD patients. Third, as previous clinical trials showed beneficial effects of NAC in combination with metformin in NASH patients (de Oliveira et al., 2008; Oliveira et al., 2019), it might be worthwhile to find other drugs for cocktail therapy with NAC.

In conclusion, to the best of our knowledge, this is the first study to explore the efficacy of NAC in NAFLD in preclinical studies through meta-analysis. We concluded that administration of NAC improved histological parameters, dyslipidemia, liver injury, insulin resistance, inflammation, and oxidative metabolism in NAFLD-induced rodents. In addition, we provided possible working mechanisms of NAC by systematically reviewing the literature. Finally, we offered a comprehensive transcriptomic analysis that may support and highlight our findings. The evidence presented in this study may be a headstone for future NAFLD clinical trials with NAC.

## Figures and Tables

**Table:** 

**ACSL1**	acetyl-coA carboxylase 1
**ALT**	alanine aminotransferase
**ARRIVE**	animal research: reporting of *in vivo* experiments
**AST**	aspartate aminotransferase
**CAT**	catalase
**CI**	confidence interval
**DGAT**	diacylglycerol acyltransferase
**ER**	endoplasmic reticulum
**FAT**	fatty acid translocase
**FFA**	free fatty acid
**G-CSF**	granulocyte colony-stimulating factor
**GGT**	gamma-glutamyl transferase
**GPx**	glutathione peroxidase
**GR**	glutathione reductase
**GSEA**	geneset enrichment analysis
**GSH**	glutathione
**GST**	glutathione S-transferase
**HDL**	high-density lipoprotein
**HFD**	high-fat diet
**HOMA-IR**	homeostatic model assessment for insulin resistance
**IL**	interleukin
**LDL**	low-density lipoprotein
**MCD**	methionine-choline deficient
**MGAT**	monoacylglycerol acyltransferase
**NAC**	N-acetylcysteine
**NAFLD**	nonalcoholic fatty liver disease
**NAS**	NAFLD activity score
**NCBI**	national center for biotechnology information
**PRISMA**	preferred reporting items for systematic reviews and meta-analysis
**RNA-seq**	RNA sequencing
**scRNA-seq**	single-cell RNA sequencing
**SD**	standard diet
**SMD**	standard mean difference
**SOD**	superoxide dismutase
**SYRCLE**	systematic review center for laboratory animal experimentation
**TC**	total cholesterol
**TG**	triglyceride
**TNF**	tumor necrosis factor
**TUNEL**	terminal deoxynucleotidyl transferase dUTP nick and labeling
**WD**	western diet

## Data Availability

The datasets presented in this study can be found in online repositories. The names of the repository/repositories and accession number(s) can be found in the article/Supplementary Material.
