# Supplementary material for: Comprehensive transcriptomic analysis and meta-analysis identify therapeutic effects of N-acetylcysteine in nonalcoholic fatty liver disease
Source: Front Pharmacol. 2023 May 15;14:1186582. doi: 10.3389/fphar.2023.1186582 (PMC10225598; doi:10.3389/fphar.2023.1186582)
Supplement: Supplementary file 1 [file DataSheet1.docx]

Supplementary Material

Comprehensive transcriptomic analysis and meta-analysis identify therapeutic effects of N-acetylcysteine in nonalcoholic fatty liver disease

Keungmo Yang^1,†^, Hee-Hoon Kim^2,†^, Young-Ri Shim^2^, Tom Ryu^3^, and Chang Wook Kim^1,*^

*** Correspondence:** Chang Wook Kim, M.D., Ph.D.: [cwkim@catholic.ac.kr](mailto:cwkim@catholic.ac.kr)

#
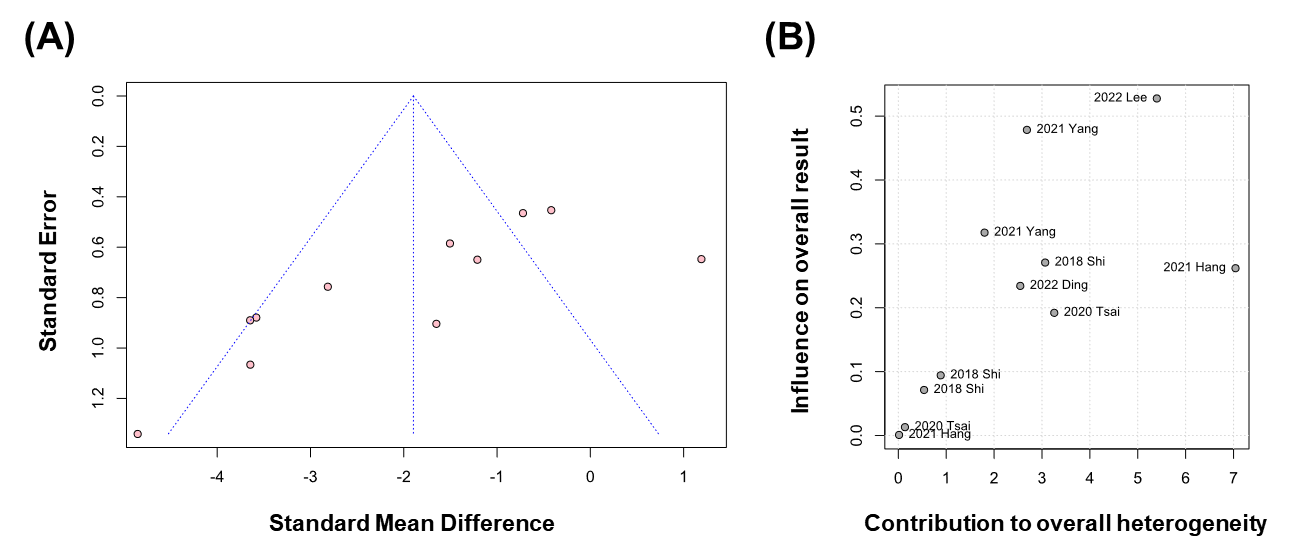
SUPPLEMENTARY FIGURES

**Supplementary Figure 1. Publication bias and sensitivity analysis for the serum triglyceride levels.** (A) Funnel plot for the publication bias. (B) Baujat plot for serum triglyceride levels of included studies.


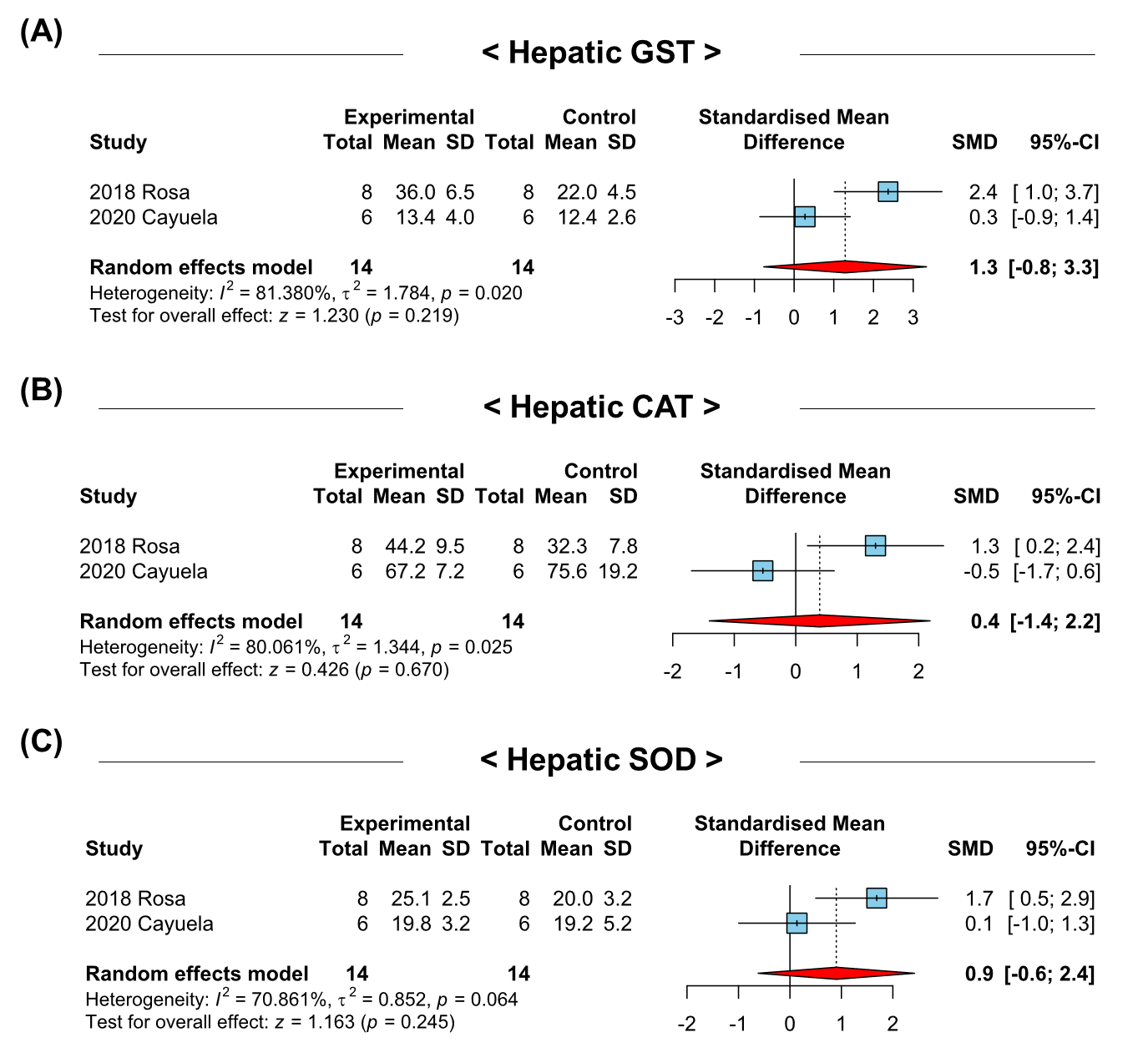


**Supplementary Figure 2. Forest plots for the effects of NAC on markers for oxidative stress in preclinical studies of NAFLD.** (A) Hepatic GST levels. (B) Hepatic CAT levels. (C) Hepatic SOD levels.


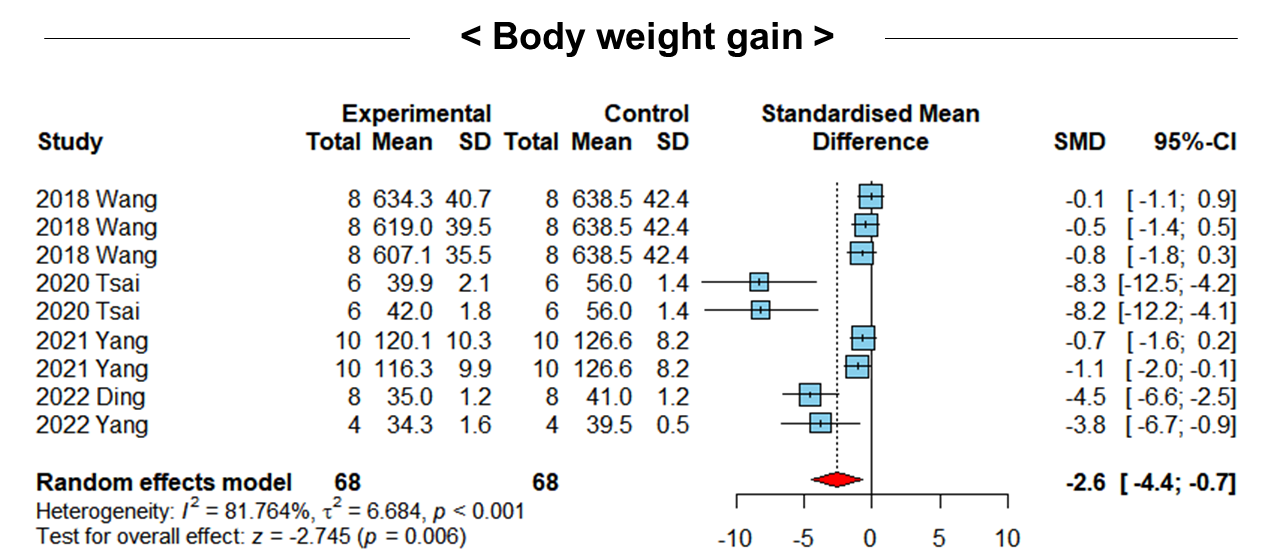


**Supplementary Figure 3. Forest plot for the effects of NAC on body weight gain in preclinical studies of NAFLD.**

# SUPPLEMENTARY TABLES

**Supplementary Table 1. Literature searching strategy**

| PubMed |
| --- |
| "acetylcystein"[All Fields] OR "acetylcysteine"[MeSH Terms] OR "acetylcysteine"[All Fields]) AND ("naflds"[All Fields] OR "nonalcoholic fatty liver disease"[MeSH Terms] OR ("nonalcoholic"[All Fields] AND "fatty"[All Fields] AND "liver"[All Fields] AND "disease"[All Fields]) OR "nonalcoholic fatty liver disease"[All Fields] OR "nafld"[All Fields]  Texts in all fields were searched.  Date of search: January 10, 2023  Result: 54 records were found. |
| Web of Science |
| 1. ((acetylcysteine) OR (acetylcystein)) word variations were searched.  2. ((nonalcoholic fatty liver disease) OR (nafld)) word variations were searched.  3. #1 AND #2  Texts in all fields were searched.  Date of search: January 10, 2023  Result: 59 records were found. |
| Cochrane Library |
| 1. ((acetylcysteine) OR (acetylcystein)) word variations were searched.  2. ((nonalcoholic fatty liver disease) OR (nafld)) word variations were searched.  3. #1 AND #2  Texts in all fields were searched.  Date of search: January 10, 2023  Result: 18 records were found. |

**Supplementary Table 2. Leave-one-out analysis for serum triglyceride levels**

| **Removed study** | **SMD** | **95 % CI** | ***P value* of effects** | ***I^2^* (%)** |
| --- | --- | --- | --- | --- |
| 2018 Shi | -0.97 | [-1.3371; -0.6100] | < 0.01 | 66.4 |
| 2018 Shi | -0.87 | [-1.2271; -0.5085] | < 0.01 | 65.9 |
| 2018 Shi | -0.83 | [-1.1858; -0.4730] | < 0.01 | 62.6 |
| 2020 Tsai | -0.85 | [-1.1970; -0.4939] | < 0.01 | 62.4 |
| 2020 Tsai | -0.95 | [-1.3021; -0.5875] | < 0.01 | 66.9 |
| 2021 Hang | -0.83 | [-1.1811; -0.4853] | < 0.01 | 55.2 |
| 2021 Hang | -0.92 | [-1.2700; -0.5676] | < 0.01 | 67.1 |
| 2021 Yang | -1.05 | [-1.4257; -0.6841] | < 0.01 | 62.8 |
| 2021 Yang | -1.03 | [-1.4011; -0.6601] | < 0.01 | 64.4 |
| 2022 Ding | -0.84 | [-1.1929; -0.4789] | < 0.01 | 63.4 |
| 2022 Lee | -1.06 | [-1.4146; -0.6988] | < 0.01 | 58.0 |

Abbreviations: CI, confidence interval; SMD, standardized mean difference.
